# Dental Education

**Published:** 1856-06

**Authors:** H. R. Smith


### The Dental Register
### OF THE WEST.

VOL. IX. JUNE 1856. NO. 4.

### Original Essays and Communications.

### DENTAL EDUCATION.

BY H. R. SMITH, M. D., D. D. S.

It has been often said, that Dentistry is a trade, and that any one who is an ingenious mechanic, will make a good operator, or in other words by a few months labor in a laboratory he will be fitted to practice dentistry.

There was a time when, to a certain extent, such remarks may have been true; for then dentistry was not considered of much importance, and any one who had strength to wrench out a tooth, no matter how roughly it was done, was permitted to practice.

And, if in the attempt, his rude and ill shaped *turnkey* should slip a score of times ere the enemy had been expelled, and several other innocent members were broken off, or knocked out, when the right one did come out, a shout of victory was uttered by the bold operator, and the patient was consoled for the loss of the well ones, by the good and philosophical reasons of the operator, "that the tooth was grown fast to the other teeth and could not be got out otherwise.

No matter if the maxilla was badly fractured, or the gums and mouth badly bruised, all could be accounted for by the same process of reasoning.

And if a tooth was accused of aching, like the victims of the Salem witchcraft, without any reasons to the contrary, it was condemned to the sacrifice. Even the physician, who ought to have been a competent operator, did not always know the anatomy of the parts upon which he operated. Nor was it considered necessary, for the operation was considered very simple and fraught with no danger to the patient. The loss of a tooth, even a front one, was considered of little consequence, and then oftentimes no charge was made for taking them out. And for mechanical dentistry, the most fit and qualified persons, even blacksmiths and watch tinkers, who had not sense and tact enough to succeed in their business, or worse, too indolent to work at it, were considered competent for dentistry, and at once turned their attention to it. Such has been, and is even now to a certain extent, the situation of our profession.

But let us for a moment review the subject, and see if such men are truly competent. We should not think of setting a blacksmith to repair a valuable watch, or a shoemaker to making jewelry. Why? Because we say this, they are not acquainted with the subject, or the machinery to be repaired. A good reason truly, and one that no sensible person will gainsay.

But at the same time we consider it right to trust a man entirely ignorant of our more delicate organs, our teeth, to go on to extract, repair and repair them at pleasure, as his duplicity may suggest. Those who consider that a mere machinist is all that is necessary to make a good operator are very much mistaken.

There are a class of operators, who have been somewhat successful in making money, and who, without the advantage of a systematic course of education, have succeeded in gaining a reputation, and they think that because they got along so well, that more education is not necessary. And we often hear of young men starting out for themselves in dentistry, who have been with these operators some three or four months, and are thoroughly qualified to practice all the various branches of Dentistry, not even for a moment suspecting that there is any one who knows more of the science than they do themselves. They unblushingly proclaim to the public their various qualifications, and ask for a liberal patronage. But question them for a few moments, and you will find, that all their knowledge consists in pulling teeth, or, getting up a partial set of artificial teeth. But of the science of the profession they know nothing. And if you ask them questions relating to chemistry, anatomy, physiology, pathology, or therapeutics, they will in answer, ask you what these subjects have to do with dentistry. Thoughts of the development of the human organs never entered their heads, and that philosophy was necessary to perform a good mechanical operation, was an idea too absurd for even a moments reflection. But if their curiosity is at all excited, and they question you still farther, explain to them, that in the first place, a knowledge of anatomy is necessary that they may know the number, name, and situation of the dental organs, how shaped, how situated, and the relation they bear to other organs. That these little pieces of bones are supplied with arteries, veins, nerves, and absorbents, and all so intimately connected with other parts of the body, that any derangement among them may affect even the most remote part. And that physiology explains the development of these organs, and the laws which govern them in a state of health, and the part they perform in carrying out the great action of the human body. Pathology will teach them that these organs may become diseased, and through them the whole system. And that they may be sympathetically affected, while the cause is in some other organ remote from them. It will also point out the landmarks by which we can trace out the cause and the effects. While therapeutics will come forth a willing servant to apply the soothing balm to restore the suffering member to its normal state. Chemistry will explain the nature of the constituents which go to make up these delicate organs, and the changes which they undergo when acted upon by foreign agents. It will also tell them what is to be used and what to be avoided in order to keep them in their original state of preservation. Also why one material may be used with impunity, while another would produce the most disastrous results. And how a systematic course of operative and mechanical dentistry, will stand forth ready to remedy any defects caused by the ravages of disease, and restore in a measure that which was lost. And lastly, how all together will teach them how to practice successfully, all the branches connected with the science of dentistry, with profit to themselves and satisfaction to their patrons.

The time was, when with few very honorable exceptions, dentists were not qualified to teach these various branches, so necessary to a scientific education. And when they *were* capable of doing it, very few indeed would take the time necessary to qualify their students in them. Hence there sprung up a necessity of some organized system of instruction, whereby students must qualify themselves to rank among professional men instead of mechanics.

Within a few years, dentistry has increased to a great extent, and with it a general intelligence of the public on dental subjects. No longer can the professional quack, pass into public favor by his flaming handbills, or paid newspaper puffs. The pretender is seen through all of his motions, and he is recompensed accordingly. More permanent and reliable credentials were called for, and the call was met by a few of the leading men in the profession. Starting a dental school under the guarantee of a State charter. A second and third school has been called for, with full boards of teachers in the various branches, and who after a rigid collegiate course, send their students forth to greet the public with sealed and attested guarantees of their qualifications. These schools are now ranked with their sister institutions, the medical colleges.

Their banners are opened to the breeze, and their colors are planted on the outer walls. They call for volunteers to enlist to carry on the contest for independence in the noble cause of dental science.

Are there not many fathers in the profession, who will say to their students when entering the profession, as did the Roman fathers to their sons when sending them to battle, "Go, and return bearing on your shields, either victory or death!"

It is for those who are now educating students for the profession, to set high the standard of professional excellence. Let them come up manfully and sustain Dental Colleges, discountenance quackery and ignorance whenever seen in our profession, and strive to make competent and honorable operators. By so doing they will not only bring honor on themselves as instructors, but do an invaluable service to the public at large.

But how many of our dentists are doing this? How many are advising their students to take a thorough systematic collegiate course of study, before entering upon the duties of the profession? How many are willing even by a kind word to forward the enterprise? And not for Dental Colleges alone; but how many are there, who are disposed to aid dental science by encouraging Dental Associations? Out of the vast number of dentists in the United States, how many have enrolled their names in a Dental Association of any kind? How many take the dental periodicals, or even one of them? How many of that number can tell you the composition of a tooth, why acid would destroy it, or why gold as a filling would preserve it better than a baser metal? How many could, if in extracting a tooth, they should rupture an artery, and simple remedies fail, save the life of their patient by putting a ligature on the bleeding vessel? And lastly, how many in that goodly number can perform one half of the operations coming under the hands of the dentist, without committing many gross errors of mal-practice.

Let the private influence,—the daily street talk—the newspaper advertisements—and the bad operations which daily meet our eyes, answer some of these questions. Let the future show who are for, and who against dental progress and improvement.

The sun has arisen on our profession, and dispelled many of the clouds that hung over it, and by the aid of science the darkness is disappearing, and a glorious day may crown our efforts. All that is now wanted, is united action in our profession to push on the car of improvement. A willingness to aid in all efforts to elevate the profession, to sustain our Dental Colleges, and dental periodicals, and form and sustain Dental Associations. Then we may be ranked as truly among the benefactors of mankind.

